# The existential realities of dancing

**DOI:** 10.3389/fcogn.2024.1372945

**Published:** 2024-06-13

**Authors:** Maxine Sheets-Johnstone

**Affiliations:** Department of Philosophy, College of Arts and Sciences, University of Oregon, Eugene, OR, United States

**Keywords:** coordination dynamics, qualitative dynamics, wellbeing, mindful bodies, brain

## Abstract

Can we again learn about ourselves and our surrounding world through dance as we age, thereby promoting our own health? This article documents facts of life showing that “older adults” do not have to learn to be cognitive of their movement, affective dispositions, or surrounding world; they have been experientially cognitive of all by way of tactility, kinesthesia, and affectivity from the beginning. Present-day cognitive neuroscience, concentrating and theorizing as it does on the brain's neuroplasticity, is however deficient in recognizing these experiential realities. Research studies on the brain and behavior, in contrast, demonstrate that coordination dynamics are the defining feature of both neurological and kinesthetic coordination dynamics. These dynamics are central to corporeal concepts, to the recognition of if–then relationships, and to thinking in movement. In effect, the brain is part of a whole-body nervous system. The study proceeds to show that the qualitative dynamics of movement that subtend coordination dynamics are basic to not only everyday movement but also to dancing—to experiencing movement kinesthetically and to being a mindful body. When Merce Cunningham writes that dance gives you that “single fleeting moment when you feel alive” and is not for “unsteady souls” and English writer D. H. Lawrence writes that “[w]e ought to dance with rapture that we are alive, and in the flesh, and part of the living incarnate cosmos,” their words are incentives to those who are aging to awaken tactilely, kinesthetically, and affectively to the existential realities of dance.

Can we again learn about ourselves and our surrounding world through movement as we age, thereby promoting our own health? In other words, can we learn by moving and being attuned tactilely and kinesthetically to our movement as we grow down just as we learned by moving and being so attuned when growing up? To answer this question and specify the relation between health in aging and not just moving but also dancing, several clarifications are necessary and warrant attention.

## Clarifications: facts of life and their relevance

The idea that dancing and “embodied cognition” are separate human abilities properly and commonly studied in separate discipline research programs that must thus be shown to be conjoined does not accord with experience. Dancing awakens experienced existential realities not of *having a body* but of *being a body*. It awakens a tactilely, kinesthetically, and affectively attuned body experiencing its own movement; thus, a mindful body directly and immediately feels the qualitative dynamics of its movement, whether when reaching for a glass, running to greet a friend, throwing the covers off the bed, trimming a hedge, or dancing. Kinesthesia and tactility are ever-present sensory faculties of living and lived bodies. They do not turn on and off like a switch. Thus, to begin, realizing that the duality of dancing and cognition is imposed is important; like the body and the brain and the mind and the body, the duality of dancing and cognition, even dancing and “embodied cognition,” is not a rock-bottom, experientially natural state of being. On the contrary, it is a fabricated state of being, an inaccurate description of living and lived bodies— both human and non-human.

Animate forms of life do not come into the world as mere moving bodies that must *learn* to be cognitive of their own movement and the immediate world around them that they, in fact, feel directly by always being tactilely and kinesthetically connected with it in both movement and stillness from the start. Barring pathological conditions, human infants are indeed already naturally attuned to cognitions, cognitions not in need of “embodiment” for they are already bodily formed and tethered, both tactilely and kinesthetically, and, in fact, affectively, for example, when stretching, kicking, crying, and feeling tears rolling down cheeks, or lying quietly in a crib.

The preceding developmental facts of life highlight the fact that the belief, idea, or claim that dancing and cognition are basically separate human abilities does not accord with experience. In effect, just as infants do not come into the world as mere moving bodies that must learn to be cognitive, so “the elderly” do not enter the world of dance as mere moving bodies that must learn to be cognitive of their movement or affective disposition to move or be cognitive of the immediate world about them that which they have been directly connected to tactilely, kinesthetically, and affectively from the start. Older adults are indeed not a new hominid species or a diminished form of the species *Homo sapiens sapiens*. However oblivious, non-attentive, and even disdainful they might have been or even now are of their bodies, older adults, like infants, are nevertheless naturally attuned to cognitions that do not need validation or sanctification as a “brain event,” for cognitions are naturally experientially formed and tethered in tactilely, kinesthetically, and affectively felt and perceiving bodies in both their movement and stillness. In fact, the brain of any animate form of life is part of *a whole-body nervous system* and thus is an integral part of an individual's anatomical, physiological, and existential being and, furthermore, an individual being the individual that it is.

One might credibly judge that some of “the elderly” have lost their awareness of themselves as *being a body* and thus lost a tactile-kinesthetic awareness and an attentiveness to the flow of their movement as they move, with their bodily awareness now tethered simply to their being unable to move in their earlier customary and even routine ways and not being able to do certain things. In effect, the cognitions of these individuals are limited regarding movement, being commonly linked in a tunneled way with an object of some kind, be it a key, a door, a fly, or a person (as well as a diverse range of other animate forms of life), and accomplishing something with it.

Present-day cognitive science commonly interprets cognitions as brain events. Of considerable interest in this context are the comments of the prime subject of an extensive research program titled the “Interesting Brains Project.” The project focuses on differences in the structure of human brains and is written about at length in an article in *Science News* by Meghan Rosen, a staff writer of the journal. Rosen (2023) notes, for example, that some human brains “have holes in their frontal or temporal lobes; others are missing parts of their cerebellum.... Still other participants have brain matter that's squished up against the sides of their skull; scans show voids that appear to have ballooned from the brain's center” (pp. 19–20). In particular, Rosen examines what Elyse G., a prime subject of the project, points out in the opening section of an article on the Project by cognitive neuroscientist Evelina Fedorenko, which might be considered either a cautionary note, a wake-up call, or both: “Please do not call my brain abnormal, that creeps me out.... My brain is atypical. If not for accidently finding these differences, no one would pick me out of a crowd as likely to have these, or any other differences that make me unique.” Rosen later emphasizes the fact that “Elyse hopes the message [her message] comes through for doctors and research scientists, immediately quoting directly from Elyse G.'s message: “I want them [doctors and research scientists”] to understand that this is a person they're reading a paper about, not a disembodied brain in a jar” (ibid., p. 21). Indeed, Elyse's brain is not a ‘brain in a vat', as in thought experiments focused on “the brain” by earlier scientists and philosophers. Her brain is, like her liver and kidneys, her arms and legs, a living part of the whole living person that she is and, as pointed out earlier, is *part of a whole-body nervous system*.

Furthermore, in this context, research studies on *coordination dynamics* are critically important. In particular, J. A. Scott Kelso, founder and director of the Center for Complex Systems and Brain Sciences, has descriptively and informatively written in ever-enlightening ways and perspectives about how, with respect to the complexity of the brain, “[a]t each level of complexity, novel properties appear whose behavior cannot be predicted from knowledge of component processes alone. To reduce a person's behavior to a set of molecular configurations is, as English neurobiologist Steven Rose once said, to mistake the singer for the song” (1995, pp. 227–228). The discovery of Kelso spells out the basic dynamic character of the brain's coordination dynamics in an experience, namely, his reading and then following a directive printed on the Yellow Pages phone directory: “Let Your Fingers Do the Walking.” In wondering how to demonstrate the spontaneously self-organizing dynamic patterning, he let his fingers do the walking and discovered *spontaneous phase transitions*. He writes:

It is the winter of 1980 and I'm sitting at my desk in my solitary cubicle late at night. Suddenly from the dark recesses of the mind an image from an ad for the Yellow Pages crops up: ‘Let your fingers do the walking'. To my amazement I was able to create a ‘quadruped' composed of the index and middle fingers of each hand. By alternating the fingers of my hands and synchronizing the middle and index fingers *between* my hands, I was able to generate a ‘gait' that shifted involuntarily to another ‘gait' when the overall motion was speeded up.... On hindsight, the emergence of this idea was itself a kind of phase transition. (Kelso, 1995, p. 46)

As commented and elaborated elsewhere, “The *idea* of letting his fingers do the walking was a spontaneous breakthrough into a new mode of thinking about spontaneously self-organized movement. It was, in other words, an *ideational* phase transition that aptly and finely exemplifies *thinking in movement*” (Sheets-Johnstone, 1981, 1999/2011, italics added; see also Sheets-Johnstone, 2010a, 2014c). Cognitive achievements that result from thinking in movement are commonly affectively charged, as in the phrase “To my amazement.” Cognition and affectivity involve us in the world, meaning that they animate us, are foundational to our being the animate forms we are, and lead us to explore, doubt, fear, come to know, wonder, delight in, and so on, such as when, for instance, we “let our fingers do the walking.” Kelso (1995) precisely recognizes the centrality of animation—of movement—when he writes,

It is important to keep in mind... that the brain did not evolve merely to register representations of the world; rather, it evolved for adaptive action and behavior.

Musculoskeletal structures coevolved with appropriate brain structures so that the entire unit functions together in an adaptive fashion.... [I]t is the entire system of muscles, joints, and proprioceptive and kinesthetic functions plus appropriate parts of the brain that evolves and functions together in a unitary way. (p. 268)

Kelso's specification of how *metastability* undergirds coordination dynamics is informative from several perspectives. Kelso (2022) begins by defining and then succinctly describing metastability: “*metastability* (from meta meaning beyond)... is a key dynamical mechanism for understanding how interacting components engage and disengage fluidly and synergistically over time (Kelso, 1995).” (Kelso, 2022, p. 9). He then explains in fine, edifying detail (ibid.):

Metastable phase attraction between neural ensembles over multiple frequency bands has been proposed to explain how brains flexibly enter and exit coherent spatiotemporal patterns of neural activity (e.g., Bressler and Kelso, 2001; Aguilera et al., 2016; 2016; Fingelkurts and Fingelkurts, 2004; Schwappach et al., 2015). Fluid thinking, from the perspective of metastable coordination dynamics, is when brain rhythms are neither completely synchronized nor desynchronized. Instead of phase synchronized states that must be destabilized if switching is to occur, metastability consists of a subtle dwell and escape dynamic in which thinking is never quite stable and merely expresses the joint *tendency* for neural areas to synchronize together and to oscillate independently. Metastable coordination dynamics rationalizes James (1980) beautiful metaphor of the stream of consciousness as the flight of a bird whose life journey consists of “perchings” (phase gathering, integrative tendencies) and “flights” (phase scattering, segregative tendencies). Both tendencies appear to be crucial: the former to summon and create thoughts; the latter to release brain regions to participate in other acts of being, knowing, and doing (Kelso, 2008).

In sum, what many present-day neuroscience researchers invoke as the “neuroplasticity” of the brain^1^ is a matter of metastable coordination dynamics that lucidly specify and describe the shifting nature of neuronal connections and the cognitive gifts of their flexibility. In complementary ways, previous research of earlier scientists testifies to the existential import of a brain's metastable coordination dynamics and its everyday and new cognitive gifts. Based on his investigations and studies, physiological psychologist Sperry (1939, p. 295) concluded not only that the brain is an organ of and for coordinated movement but that the function of consciousness or subjective experience is also “*coordinated movement*” (Sperry, 1952, p. 309). Sperry's conclusions document the preeminence of movement in animate lives and the ability to think in movement. Moreover, neuroscientist and neurophysiologist Marc Jeannerod's conclusion regarding the sensory modality of kinesthesia testifies similarly to the preeminence of movement in animate lives and the ability to think in movement. After a lengthy examination of “conscious knowledge about one's actions” and conducting research that addressed the question of such knowledge and included experimental studies focused on pathologically afflicted individuals, Jeannerod (2006, p. 56) concluded: “There are no reliable methods for suppressing kinesthetic information arising during the execution of a movement.”

The import of moving to wellbeing, specifically to the “[promotion] of brain health in the elderly” by awakening “the neuroplasticity of the brain,” necessarily warrants taking not only the brain as neurophysiologically integral in dynamically constructive ways to whole living bodies as detailed earlier but also into account primary existential facts of life with respect to the development of the brain prenatally and in infancy (Quoted from Frontiers's announcement of this Special Issue on “Cognition and Movement”). In particular, it means taking into account the fact that, barring pathological conditions, tactility and kinesthesia are the first sensory systems to develop *in utero* and that, barring pathological conditions, we all come into the world moving. In the beginning and developmentally, our cognitions are thus not linguistically tethered but bodily tethered—most basically, tactilely and kinesthetically tethered. On this basis, we form *non-linguistic corporeal concepts*—for example, of near and far, sharp and smooth, heavy and light, and open and close. Moreover, we form *if–then relationships* based on our experiences, relationships that testify to the foundational animate ability *to think in movement*.^2^

Infant psychiatrist and clinical psychologist Stern (1985) pinpoints just such if–then relationships when exemplifying what he terms “consequential relationships,” relationships experienced by infants, for example, “when you shut your eyes it gets dark” (p. 71). In ways akin to Stern, infant psychologist Lois Bloom terms the awareness of such experiences “relational concepts.” Thus, Bloom also implicitly recognizes if–then relationships and the basic developmental phenomenon of thinking in movement (for a full description, see Sheets-Johnstone, 1999/2011). She does so when, in defining relational concepts, she states, “Children learn about relationships between objects by observing the effects of movement and actions done by themselves and other persons” (Bloom, 1993, p. 50); for example, slapping bath water causes a splash (Bloom, 1993). Furthermore, as she explicitly points out and exemplifies, relational concepts develop outside of language. They are developed based on observations of movement (Bloom, 1993, p. 50–51). Infant and child psychologist Jerome Bruner affirms this insight with his emphasis on narrative as the primary form of human discourse and the central place of action in that discourse. He writes that, when young children “come to grasp the basic idea of reference necessary for any language use... their principal linguistic interest centers on *human action and its outcomes*” (Bruner, 1990, p.78, italics in original). His point is that narrative structure is, in the beginning, concerned with movement, in particular with “agentivity” (Bruner, 1990, p. 77): “Agent-and-action, action-and-object, agent-and-object, action-and-location, and possessor-and-possession,” he states, “make up the major part of the semantic relations that appear in the first stage of speech” (Bruner, 1990, p. 78).^3^

In sum, we humans learn about ourselves and our surrounding world during our developmental years by moving, by being attuned tactilely and kinesthetically to our movement, and by reaping from it an ever-increasing cognitive awareness and expansion of our practice and ability to think in movement, in effect, to being able to navigate the world in efficient, effective, and enjoyable ways.

We may, in turn, ask: can we again learn about ourselves and our surrounding world through movement as we age, thereby promoting our own health? In other words, and as asked earlier, can we learn by moving and being attuned tactilely and kinesthetically to our movement as we grow down just as we learned by moving and being so attuned when growing up? The question should be stated more specifically, for it is not just a question of moving but of dancing. For starters, it is essential to recognize that to dance is not simply to move. To dance is to be quintessentially attuned wholly and exclusively to movement, to *being-in-movement*. Thus, in dancing, movement is not tethered to accomplishing something, fulfilling a promise or an obligation, passing a test of one's abilities, and so on. Being attuned to dancing is thus substantively, essentially, and experientially different from “movement-rich exercises for the elderly.” Exercising, including even “movement-rich” exercising, is essentially a specifically defined practice during which certain movements are performed: first this movement, then that movement, next this movement, and so on (Quoted from Frontiers's announcement of this Special Issue on “Cognition and Movement”). In short, to exercise is to move in conformity to a set series of movements rather than to experience a flow of movement moving through one, the latter being an experience in which *the qualitative dynamics of movement resonate exclusively and continuously in a felt whole-bodily sense*. The qualitative dynamics of movement are analytically described in terms of their kinesthetically felt qualities: tensional, linear, amplitudinal, and projectional—thus, for example, and respectively in experiential terms, as strong, curved, expansive, and abrupt. In effect, one's awareness is not on *doing* movements but on *being-in-movement*, i.e., on the felt dynamics of movement itself. As pointed out and elaborated elsewhere (Sheets-Johnstone, 1983, 2014a,b; see also Sheets-Johnstone, 2024a), familiar dynamics—tying a knot, brushing one's teeth, writing one's name, pulling weeds, typing, playing a Bach prelude, and so on—are woven into our bodies and played out along the lines of our bodies; they are kinesthetic/kinetic melodies in both neurological and experiential senses (Luria, 1966, 1973). Indeed, were someone else to brush our teeth, we would immediately recognize that someone else was brushing our teeth, not just because we were not holding the toothbrush and not only because we could actually see someone in front of us holding and moving our toothbrush but because we would also feel a foreign dynamics inside our mouth. In sum, when we turn attention to our coordinated dynamics (Kelso, 1995; Kelso and Engstrøm, 2006), we recognize kinesthetic melodies; they bear the stamp of our qualitatively felt movement patterns and our familiar synergies of meaningful movement.^4^

From this perspective, exercising is experientially a poverty-stricken, vapid form of moving compared to dancing. The difference is not a prejudiced theoretical claim but an existential reality, even a creative existential reality, and this is because movement, any movement, creates its own space–time–force dynamic, precisely by way of the inherent dynamic qualities of movement itself. In short, while thinking about movement as occurring *in space* and *in time* is not uncommon or, in particular, objective, movement definitively *creates its own space and time*. That it does so is what distinguishes, for instance, one person's recognizable walking style from another person's style. Phenomenological philosopher Husserl (1989) singles out just such recognizable individual differences when he writes about our understanding of others by way of their “typicalities”:

Personal life manifests a typicality, and each personal life manifests a different one. For certain periods, this typicality remains identical, even if the “experiences” (*the realm of the experiential apperceptions constantly being newly formed*) of the person grow.... [I]n conformity with this typicality,... I can say that if this person finds himself in these circumstances he will behave according to type and that if the circumstances change he will still observe the type. (Husserl, 1989, p. 284; italics in original).

Husserl, in fact, specifies typicality in terms of style:

Every man has his character, we can say, his style of life in affection and action, with regard to the way he has of being motivated by such and such circumstances. And it is not that he merely had this up to now; the style is rather something permanent, at least relatively so in the various stages of life, and then, when it changes, it does so again, in general, in a characteristic way, such that, consequent upon these changes, a unitary style manifests itself once more. (Husserl, 1989, p. 283)

It is notable and worth recognizing that Charles Darwin wrote of style and typicality in different but complementary ways when he described individual differences among animals of the same species. In particular, he described individual differences in terms of variations, variations in agility, dispositions, temperament, and alertness, for example. As pointed out elsewhere (Sheets-Johnstone, 2022, pp. 2–3),

Darwin begins Chapter I of *The Origin of Species* titled “Variation under Domestication” with the following observation: “When we look to the individuals of the same variety or sub-variety of our older cultivated plants and animals, one of the first points which strikes us, is, that they generally differ much more from each other, than do the individuals of any one species or variety in a state of nature (Darwin, 1968 [1859], p. 71). In Chapter II, titled “Variations under Nature,” he writes, “No one supposes that all the individuals of the same species are cast in the very same mold. These individual differences are highly important for us, as they afford materials for natural selection to accumulate, in the same manner as man can accumulate in any given direction individual differences in his domesticated products.” (Darwin, 1968 [1859], p. 102)

In short, humans vary individually just as all animals within the phylum Chordata and subphylum Vertebrata do. A further fact is relevant in this context, namely, the foundational importance of movement. As noted earlier, we, and other forms of animate life, come into the world moving. Movement is indeed our mother tongue: we are movement born and remain animate until we die. From an evolutionary perspective, as well as cultural and social perspectives, animate forms of life survive and reproduce by virtue of their movement—their kinetic ability to find food, their agility in fighting and avoiding predators, their concentrated and full-bodied pursuit of a mate, and, with respect to some forms, their diverse ministrations in raising young, not to mention the ability of the young to learn “how to” from their elders. It is thus hardly surprising that kinesthesia and tactility—and the earlier proprioceptive form of movement sensitivity and awareness in invertebrates by way of tactility (Lissman, 1950; Laverack, 1976; Mill, 1976; see also Sheets-Johnstone, 1999/2011)—are the first sensory systems to develop. Animate forms of life are basically tactile-kinesthetic bodies.

Another essential fact of life not only is significant but also warrants emphasis. The current practice, and even fad, of separating “the brain,” notably the human one, from the body is a breach of anatomy and neurophysiology. The brain, whether of a human or any animate form of life, is indeed not a brain in a vat, a distinct and wholly separate organ, or a structured independent container or bin that functions completely on its own with no outside connections, influences, or other bodily references. In particular, and as emphasized earlier, a brain, whether of a human or any animate form of life, is part of *a whole-body nervous system*, and its afferent and efferent neural connections are essential and indispensable parts of its structure and functions. Moreover, while studying and analyzing the brain is substantively edifying, whether of human or non-human forms of animate life, it is neither valid nor truthful to make experiential ascriptions to the brain as neuroscientists Crick and Koch (1992) do, for example, when they state that the brain “infers”: “If you see the back of a person's head, the brain infers that there is a face on the front of it” (p. 153); philosopher Flanagan (1991) does when he states that the brain “anticipates”: “Overall, our brain is the most powerful anticipation machine ever built” (p. 319); and neurobiologist Zeki (1992) does when he states that the brain “ascertains”: “An object's image varies with distance, yet the brain can ascertain its true size” (p. 69). The warning that Darwin gave in his *Notebooks* in which he recorded his research studies, questions, observations, and so on, is of seminal interest and should be given pointed consideration by those who do research on the brain. Based on his global observations of animate life and his thoughtful investigations and considerations of the research and writings of others, Darwin (1987) wrote, “Experience shows the problem of the mind cannot be solved by attacking the citadel itself—the mind is [a] function of [the] body—we must bring some *stable* foundation to argue from” (Darwin, 1987, p. 564; italics in original). As pointed out elsewhere (Sheets-Johnstone, 2010b, 2023), what Darwin meant by the words “experience shows” may be interpreted in two possible ways:

He may have been referring to philosophers who attempt to show the nature of mind “by attacking the citadel itself” [an interpretation that may, of course, be extended to present-day scientists, many of whose “attacks on the citadel itself” include experiential ascriptions to “the brain,” as exemplified above]. But, Darwin may also very well have been referring to his own extensive, highly detailed first-person experiences of animate life, experiences that showed him in person that the mind was not something distinct from the body but precisely, as he states, a function of body. In effect, animate bodies are mindful bodies.

Taking Darwin's cautionary note seriously, we may proceed to take up the question of dance, specifically addressing how dancing is a whole-body enterprise, that is a testimony to the existential reality of *mindful bodies* that move and that, in moving, create wholly qualitative dynamic forms that unfold continuously and coherently, sustained by their own integral dynamic wholeness. Indeed, the “*stable* foundation” showing that “the mind is function of body” is movement, the movement of mindful bodies. “What!” one might exclaim. “How can movement be a *stable* foundation? It won't stay still!”

## Existential realities

Husserl (1989) wrote of the unity of “body and soul,” describing and exemplifying their unity and summing up it as follows: “Man, in his movements, in his action, in his speaking and writing, etc., is not a mere connection or linking up of one thing, called a soul, with another thing, the Body. The Body is, as Body, filled with the soul through and through. Each movement of the Body is full of soul, the coming and going, the standing and sitting, the walking and dancing, etc.” (p. 252). To dance is indeed to engage body and soul in creating a dynamic form. In dancing the dance, the dancer and the dance are one. As discussed elsewhere (Sheets-Johnstone, 2024b),

The aesthetic unity of dancer and dance is indeed unique. Merce Cunningham eloquently captures the ontological, even metaphysical nature of their oneness from the point of view of the dancer: “You have to love dancing to stick to it. It gives you nothing back, no manuscripts to store away, no paintings to show on walls and maybe hang in museums, no poems to be printed and sold, nothing but that single fleeting moment when you feel alive. It is not for unsteady souls.” (Cunningham, 1968, n.p.)

In light of this unity, one may readily ask several questions with respect to dance and the elderly: Are older people ‘steady enough souls' to engage in dance? If dancing gives you nothing but “that single fleeting moment when you feel alive,” how does dancing possibly tie in with cognition and, in particular, the cognition of the elderly? Further still, how does “that single fleeting moment when you feel alive” promote brain health in the elderly?

We might begin to answer these questions by affirming first that feeling alive can be a remarkable existential experience, one that warrants recognition. The experience is perhaps the most foundational existential experience one can have. English writer Lawrence (1932) captures the affective import of that foundational existential experience in his eloquent appreciation of being alive:

“[T]he magnificent here and now of life in the flesh is ours, and ours alone, and ours only for a time. We ought to dance with rapture that we should be alive and in the flesh, and part of the living incarnate cosmos” (Lawrence, 1932, p. 200). Moreover, Lawrence affirms the authenticity of this temporally foundational fact of life by way of the living body as subject: “That I am part of the earth, my feet know perfectly” (Lawrence, 1932). Can older and elderly people affirm this fact of life—or is it beyond them? Lawrence does not mention any age limits in what he writes of the experience of being alive. Neither does Merce Cunningham mention any age limits in dancing, though we might certainly ask, as above, whether elderly people are “unsteady souls” and thus incapable of dancing and experiencing the “single fleeting moment when you feel alive.”

The import of moving to wellbeing, specifically to the “[promotion] of brain health in the elderly” by awakening “the neuroplasticity of the brain,” earlier took us back to infancy and the realization that our cognitions were originally bodily, not linguistically, tethered. The body is thus not a piece of equipment that lets us get about in the world; it is a source of knowledge. Furthermore, it is the anchor point of agency, of our ability to move and do or not move and do. As discussed elsewhere (Sheets-Johnstone, 1999/2011), Stern's (1985) experimental neonatal evidence of volition, that is, of agency, evidence based on the kinetic doings of identical infant twins, accords in fundamental ways with what Husserl describes as ‘I govern', a basic dimension of “my animate organism” (Husserl, 1973, p. 97).^5^ It testifies empirically to a psychophysical unity in the form of a tactile-kinesthetic body and, in the most basic metaphysical sense, to a kinetic tactile-kinesthetic being, a *Da-bewegung*.^6^ It clearly does not accord with the sense of self as a “mental thing”, an entity locked inside a head—or brain.

The research Stern et al. conducted involved conjoined twins who, before their separation at 4 months, were joined between their navels and sternums. The experiment involved determining any difference in the bodily movement of the twins when deterred from sucking their or their twin's fingers. When the researcher pulled one twin's arm away from her mouth, resulting in her fingers being pulled out of her mouth, the other twin resisted the pull; that is, *she attempted to pull her arm back toward herself* . When the researcher pulled the other twin's arm away from her mouth, resulting in her fingers being pulled out of her mouth, the first twin *strained forward with her head in pursuit of the retreating fingers*, i.e., in pursuit of something *alien* to her body. In documenting two distinctive bodily movements, the experiment documents a bodily sense of self in the form of a tactile-kinesthetic body that can move and move in self-directed ways, hence as a self-directed agent. In particular, it documents the volitional movement that springs from a knowing body that experiences both its movement and the surrounding world, which it feels directly. Such a body has the anatomical, neurophysiological, and existential capacity to respond perceptively and cognitively from head to toe. As such a body grows and matures, it does not outgrow its capacities as a tactile-kinesthetic body and a volitional agent unless medical and, in particular, geriatric conditions intervene and preclude the viability of these former capacities.

The health value of dancing is a life-long value that is not just intimately but also integrally tied to the capacities of the tactile-kinesthetic body and its volitional movement, that is, to the capacities of a whole-body neuromuscular system from head to toe, capacities that anchor a dynamic flow of movement and thereby attest to the aliveness of a “steady soul” experiencing that “fleeting movement of aliveness” that is the hallmark of dance and within the capacity of any and all elderly who are alive and want to feel their aliveness.

## Data availability statement

The original contributions presented in the study are included in the article/supplementary material, further inquiries can be directed to the corresponding author/s.

## Author contributions

MS-J: Writing – original draft.

## Footnotes

^1^The following are two examples of neuroplasticity in present-day neuroscience research: “What is theorized to occur is that when the presynaptic neuron stimulates the postsynaptic neuron, the postsynaptic neuron responds by adding more neurotransmitter receptors, which lowers the threshold that is needed to be stimulated by the presynaptic neuron.... Aging and neurodegenerative diseases have been associated with a decrease of neuromodulators and may contribute to a reduction in the ability of synaptic plasticity.... The theory of synaptic plasticity has also grown to include more of the evolving complexity of synaptic communication” (Puderbaugh and Emmady, 2023). “Since the brain was found to be somehow flexible, plastic, researchers worldwide have been trying to comprehend its fundamentals to better understand the brain itself, make predictions, disentangle the neurobiology of brain diseases, and finally propose up-to-date treatments. Neuroplasticity is simple as a concept, but extremely complex when it comes to its mechanisms. This review aims to bring to light an aspect about neuroplasticity that is often not given enough attention as it should, the fact that the brain's ability to change would include its ability to disconnect synapses. So, neuronal shrinkage, decrease in spine density or dendritic complexity should be included within the concept of neuroplasticity as part of its mechanisms, not as an impairment of it.... Therefore, we propose to break down neuroplasticity into two sub-concepts, “upward neuroplasticity” for changes related to synaptic construction and ‘downward neuroplasticity' for changes related to synaptic deconstruction” (Diniz and Crestani, 2023).

^2^To preface the following discussion, we should note that *thinking in movement* is integral to dancing, learning to dance, and, later, learning and performing a particular dance. To dance is indeed not doing this movement now and then this movement but allowing a continuously moving dynamic form to move through you.

^3^For further in-depth studies and probing essays on infants by a variety of dynamic systems researchers, see Smith and Thelen (1993) and Thelen and Smith (1994).

^4^For more on synergies of meaningful movement, see Sheets-Johnstone, 2012.

^5^There is no doubt that Husserl's (1973) animate organism is kinesthetically based: “Touching kinesthetically, I perceive “with” my hands; seeing kinesthetically, I perceive also “with” my eyes; and so forth; moreover I can perceive thus at any time. Meanwhile, the *kinesthesias* pertaining to the organs flow in the mode “I am doing,” and are subject to my “I can”; furthermore, by calling these kinesthesias into play, I can push, thrust, and so forth, and can thereby “act” somatically—immediately and then mediately” (p. 97).

^6^Through the methodological practice of phenomenology, Husserl identifies a further dimension of being an animate organism, namely, being a psychophysical unity: “if I reduce *myself* as a man, I get “*my animate organism*”” and “*my psyche*,” or myself as a *psychophysical unity*—*in the latter, my personal Ego*, who operates in this animate organism, and “by means of” it, in the *external world*, who is affected by this world, and who thus in all respects, by virtue of the continual experience of such unique modes of Ego- and life-relatedness, is constituted as psychophysically united with the animate corporeal organism” (Husserl, 1973, p. 97–98).
